# Improving CO_2_ Removal Efficiency with Bio-Cellulose Acetate: A Multi-Stage Membrane Separation Approach

**DOI:** 10.3390/polym17020224

**Published:** 2025-01-17

**Authors:** Attaso Khamwichit, Kamontip Wongsuwan, Wipawee Dechapanya

**Affiliations:** 1 Biomass and Oil Palm Research Center of Excellence, Walailak University, Thasala, Nakhon Si Thammarat 80160, Thailand; kattaso@mail.wu.ac.th; 2 Department of Chemical Engineering, School of Engineering and Technology, Walailak University, Thasala, Nakhon Si Thammarat 80160, Thailand; 3 Engineering Graduate Program, Walailak University, Thasala, Nakhon Si Thammarat 80160, Thailand; kamontip253925@gmail.com

**Keywords:** multi-stage membrane separation, value-added bio-membranes, cellulose acetate membrane, CO_2_ removal, biogas upgrading

## Abstract

In this comprehensive investigation, the sustainable production and utilization of gas separation membranes derived from coconut water (CW) waste was investigated. The research focuses on the synthesis of bacterial cellulose (BC) and cellulose acetate (CA) membranes from CW, followed by a thorough analysis of their characteristics, including morphology, ATR-FTIR spectroscopy, tensile strength, and chemical composition. The study rigorously evaluates membrane performance, with particular emphasis on CO_2_/CH_4_ selectivity under various operational conditions, including pressure, membrane thickness, and number of stages. The application of these membranes in gas separation units was optimized for CO_2_/CH_4_ separation performance and eco-efficiency through a multi-stage membrane approach. The findings indicate that in double-stage configurations, CA membranes with a thickness of 0.04 mm, operating at 0.28 MPa, achieve a CO_2_/CH_4_ selectivity of 35.52, significantly surpassing single-stage performance (selectivity: 19.72). Furthermore, eco-efficiency analysis reveals optimal performance at 0.04 mm thickness and 0.175 MPa, reaching 3.08 CO_2_/CH_4_ selectivity/THB. These results conclusively demonstrate the viability of converting agricultural waste into high-performance gas separation membranes, representing a significant advancement in sustainable membrane technology. This research contributes valuable insights to the field and paves the way for further innovations in eco-friendly membrane production and application.

## 1. Introduction

As the world seeks renewable energy to mitigate climate change’s impacts, innovative carbon dioxide-removal technologies have become increasingly vital. Biogas has emerged as a promising alternative, offering sustainable solutions for energy production while simultaneously addressing waste management issues. The standard composition of biogas typically consists of approximately 55–65% CH_4_ and 30–45% CO_2_ [1]; however, effective CO_2_ removal is essential for enhancing biogas’ energy yield and quality. Furthermore, effective capture and utilization of CO_2_ generated during biogas production remains a significant challenge that necessitates innovative strategies. Biogas upgrading transforms CH_4_ into renewable biomethane, while carbon capture reduces CO_2_ emissions from industrial sources. Both gases are potent contributors to global warming [2]. Improving separation technologies helps reduce industries’ carbon footprints, aligning with global climate goals. Therefore, advancements in CO_2_ and CH_4_ separation are essential for optimizing industrial processes and addressing environmental challenges [3].

The separation of CO_2_ from CH_4_, vital in industries like natural gas purification and biogas upgrading, faces technical challenges due to the gases’ similar molecular sizes. Traditional methods, such as cryogenic distillation and absorption, are energy-intensive and costly, making them less viable for large-scale use [4]. Additionally, these processes require substantial infrastructure and maintenance. To overcome these challenges, membrane-based technologies offer a more energy-efficient alternative, operating at lower temperatures and pressures and selectively separating gases [5]. However, current membrane technologies still struggle with limited capability on selectivity, durability, and scalability. Research is focused on developing more efficient, environmentally friendly membranes that reduce energy consumption and emissions [6]. While CO_2_/CH_4_ separation remains complex, membrane-based innovations hold promise for improving efficiency and sustainability. The use of CW as a carbon source in BC production could introduce lignin-like compounds into the resulting material. CW, derived from a plant source, naturally contains a variety of plant-based compounds, including sugars, proteins, and trace amounts of lignin or lignin-like substances [7]. Although *A. xylinum* synthesizes cellulose that is typically free of lignin, residual plant compounds from the CW could be carried over during the fermentation process, leading to elevated lignin measurements in the final BC product. This issue can be particularly relevant in cases where the juice has not been fully purified or treated before use, potentially affecting the purity of the BC.

Despite the growing interest in bio-based membranes for CO_2_/CH_4_ separation, significant research gaps remain, particularly in understanding how bio-based materials like BC and CA perform under varying operational conditions. Current research has demonstrated the potential of these materials for gas separation, but there are limited data on their long-term performance, scalability, and behavior under industrially relevant pressures and temperatures [8]. Moreover, while some studies have explored the fundamental properties of BC and CA membranes, their performance across different membrane thicknesses and separation stages, as well as the trade-offs between permeability and selectivity, is not fully understood [9].

This knowledge gap is especially important for organizations seeking to deploy bio-based solutions that address both environmental and economic concerns. This research seeks to fill these gaps by concentrating on a few critical objectives. This study explores the innovative use of bio-cellulose acetate as a membrane material, focusing on its ability to improve CO_2_ removal efficiency through a multi-stage separation approach. For process parameter modeling and optimization, Response Surface Methodology (RSM) is frequently used. RSM helps with understanding experimental factors, in addition to optimization. Screening, characterizing, and optimizing are the three operating modes that the software provides. Bio-cellulose acetate, derived from renewable resources, offers distinct advantages over conventional materials, including sustainability, cost-effectiveness, and superior permeability properties. By investigating the performance of this bio-based membrane in a multi-stage configuration, this research aims to provide insights into the optimization of membrane design for enhanced gas separation processes, potentially leading to improved CO_2_/CH_4_ separation efficiency that is cost-effective and aligns with environmental sustainability goals.

## 2. Materials and Methods

### 2.1. Materials

The bacterial strain *A. xylinum* was procured from the Thailand Institute of Scientific and Technological Research (TISTR) in Bangkok, Thailand. This strain was subsequently employed for standard plate count (SPC) analyses, following established microbiological protocols. CW discharged from coconut milk manufacturing was acquired from a local coconut milk producer. High-purity ammonium sulfate [(NH_4_)_2_SO_4_], with a purity exceeding 99.99%, was obtained from QRec (Bangkok, Thailand) for use in this study. Ethanol (96%) was obtained from Vdells Siam, Bangkok, Thailand, and sugar was supplied by Mitr Phol Sugar Corporation Ltd., also based in Bangkok, Thailand. Additionally, a 5% aqueous acetic acid solution was supplied by Tang Sang Hah Co., Ltd., Samutprakan, Thailand.

### 2.2. Experimental Procedure

To ascertain the ideal parameters impacting both eco-efficiency and CO_2_/CH_4_ selectivity at the same time, the optimization mode was employed in this investigation. In the Differential Evolution (DE) algorithm, two key performance indicators were established for maximization: CO_2_ selectivity and eco-efficiency. Consequently, the optimization function within the DE framework was designed to identify the optimal conditions that yield maximum values for both of these critical parameters. This approach ensures that the algorithm effectively balances the dual objectives of environmental sustainability and process efficiency in determining the most favorable operational settings. The cultivation of BC from CW was conducted using the central composite design (CCD), leveraging optimal conditions identified in previous research [10,11]. The experimental conditions for all runs, as suggested by the software, included variations in pressure (from 0.1 to 0.25 MPa), thickness (from 0.03 to 0.05 mm), and the number of stages in the separation unit (1 or 2 stages). During the optimization process, both CO_2_ selectivity and eco-efficiency were maximized simultaneously.

### 2.3. BC and CA Membrane Preparation

**Figure 1:**
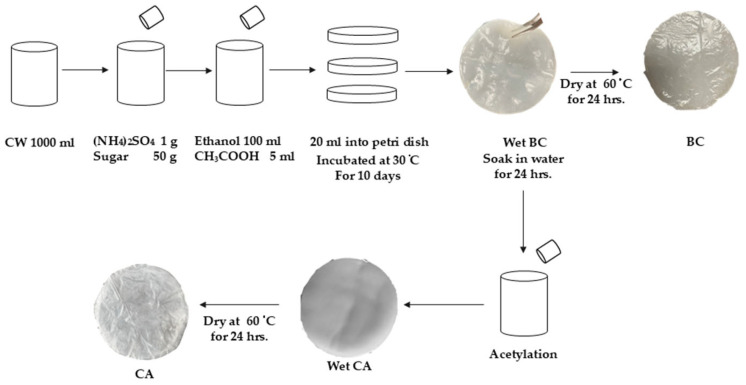
BC and CA membrane preparation.

To prepare BC and CA membranes, the procedure, as displayed in Figure 1, following the previous work [12], was performed. CW was filtered and stored at 6 °C in a sterilized container. A mixture for cultivating *A. xylinum* was prepared using 1000 mL of CW, agar powder, sugar, ammonium sulfate, ethanol, and acetic acid. After homogenization and autoclaving at 121 °C, the mixture was cooled and transferred under aseptic conditions. After seven days, colony-forming units were evaluated using the pour plate technique. The coconut substrate formulation was prepared with precision, incorporating 1000 mL of filtered CW, 1 g of ammonium sulfate, 50 g of sugar, 100 mL of 40% ethanol (*v*/*v*), and 5 mL of acetic acid. The mixture was subsequently dispensed into sterilized Petri dishes (diameter of 8 cm), with each plate receiving a standardized 20 mL aliquot of the substrate. To activate the cultivation process, 10 mL of *A. xylinum* inoculum, derived from a previous cultivation cycle, was introduced to each plate. This rigorous protocol ensures optimal growth conditions and reproducibility in BC production, adhering to established best practices in microbial cultivation techniques. The substrate was then incubated in Petri dishes under optimal conditions. These conditions were kept at 30 °C and pH 6.0 for 10 days because they were empirically determined to be ideal for BC synthesis from CW. The BC pellicle that developed on the surface of the substrate after incubation was removed and soaked for 24 h at ambient temperatures in distilled water. The BC specimen underwent a desiccation process in a laboratory-grade oven maintained at 60 °C for 24 h to ensure ample elimination of residual moisture. Following this dehydration procedure, the mass of the dried BC sample was precisely measured using an analytical weight measure to determine the quantitative yield of the synthesis BC.

The synthesized BC proceeded to undergo a semi-acetylation process to generate a bio-cellulose acetate (bio-CA) membrane. The degree of acetylation was contingent upon two primary factors: individual cellulose crystallites’ reactivity and the fibers’ accessibility are crucial factors in understanding and optimizing cellulose-based processes. Following the extraction of the BC pellicle from the growth substrate, it was immersed in distilled water and maintained at ambient temperature for 72 h. A meticulous purification method was used to remove any bacterial cell debris. Initially, the BC was exposed to a three-hour boiling treatment in 40 mL of 2% sodium hydroxide solution at 60 °C. Following this, 40 mL of 40% ethanol was added to the BC, and it was boiled for three more hours at the same temperature rate. The BC was then dried up in a controlled atmosphere at 60 °C for three hours. The last semi-acetylation phase entailed treating the BC with a precise mixture of acetic acid for 40 mL, toluene for 50 mL, and 60% perchloric acid for 0.2 mL. This comprehensive procedure ensures the effective removal of cellular contaminants, resulting in a purified CA product suitable for further analysis or application. The purification of CA was performed through a meticulous and comprehensive process to eliminate all chemical residues and impurities. The protocol involved immersing the material in 40 mL of a 75% ethanol solution for a precise duration of 2 h. Following this, the CA underwent a thorough rinsing procedure utilizing distilled water to ensure the complete removal of any remaining ethanol. The final phase of the purification process entailed drying the CA in a hot-air oven (Memmert GmbH & Co., Ltd., KG, Bangkok, Thailand) at a carefully regulated temperature of 60 °C for a prolonged period of 8 h to obtain the CA-0.05 membrane. This rigorous methodology guarantees the highest level of purity for the CA, rendering it suitable for subsequent applications requiring stringent quality standards.

### 2.4. Membrane Characterizations

#### 2.4.1. Morphology Properties

A scanning electron microscope was used to analyze the BC and CA membranes’ microstructure (SEM, Model Merlin Compact, Carl Zeiss Co., Ltd., Bangkok, Thailand). Initially, the BC and CA membranes were dried in a hot-air oven (Model FD115; Franz Binder GmbH & Co., Ltd., Bangkok, Thailand) at 60 °C for 24 h. Upon completion of the drying process, the membranes were transferred to a desiccator (Model RT-48C; Eureka Design Co., Ltd., Pathum Thani, Thailand) at ambient temperature. The dried BC and CA membranes were then mounted on carbon stubs and subjected to gold sputtering. The samples were subsequently analyzed using a Jeol Jx A-840 SEM (Jeol, Peabody, MA, USA). To obtain further details about both the BC and CA membranes’ nanostructure, SEM images were analyzed with Microsun (Version 2.0) 2000/s image analysis software. A magnification of 10,000x with an accelerating voltage of 5 kV was utilized for the analysis.

#### 2.4.2. Attenuated Total Reflection–Fourier Transform Infrared (ATR-FTIR) Spectra Analysis

ATR-FTIR spectrometer, the Tensor 27 model, produced by Bruker in Germany, was employed to accurately identify and characterize the functional groups present within the BC and CA membrane structures. The spectral analysis was performed across a wavelength range of 400–4000 cm^−1^, ensuring a thorough examination of the membranes’ chemical functional groups.

#### 2.4.3. Tensile Stress

The tensile stress properties of the synthesized BC and CA membranes were evaluated using a high-precision tensile stress machine (model DSS-10T; Shimadzu, Kyoto City, Japan). The assessment was conducted following ASTM D638 standards [13] to ensure reliability and reproducibility of results. Membrane samples with three distinct thickness levels (0.03, 0.04, and 0.05 mm) and a uniform breadth of 40 mm were securely positioned in the machine’s clamps. To achieve CA membranes with varying thicknesses, the drying duration was extended to 9 h for CA-0.04 and 10 h for CA-0.03. This precise adjustment in drying time allows for the controlled fabrication of membranes with specific dimensions. A circular CA membrane with a diameter of 8 cm has a surface area of 50.24 cm^2^. To establish statistical significance and account for potential variability, six replicate samples were subjected to incrementally increasing tensile tension until material failure occurred. This rigorous testing protocol allowed for a comprehensive analysis equation of the membranes’ mechanical properties across varying thicknesses, providing valuable insights into their structural integrity and potential applications.

#### 2.4.4. Composition of Membrane Analysis

The compositional analysis of BC and CA membranes was conducted utilizing advanced lignocellulose characterization techniques. This comprehensive examination focused on the quantification of cellulose, lignin, and hemicellulose, which constitute the primary structural components of these membrane materials. The extraction process involved heating 80 mL of acetone to 90 °C in a Soxhlet extractor for one hour, followed by a 30-min extraction at ambient temperature. Hemicellulose analysis was conducted by heating NaOH-filled test tubes to 80 °C for 210 min. Subsequently, the BC residue was filtered employing Whatman No. 1 filter papers and dried at 105 °C for three hours. For lignin analysis, 0.2 g of BC and 3 mL of H_2_SO_4_ were heated to 30 °C for one hour in a 500 mL flask. Following the addition of 56 cm^3^ of deionized water, the mixture was autoclaved at 121 °C for 15 min. The resulting solution was then filtered and dried at 80 °C for three hours. The composition of BC and CA membranes was then calculated using Equations (1)–(3) [14].

Cellulose content:

*C* (%) = 100 − *H* (%) − *L* (%) (1)

Hemicellulose content:

*H* (%) = (*W*_1_ − *W*_2_)/*W*_1_ × 100% (2)

Lignin content:

*L* (%) = (*W*_4_/*W*_3_) × 100% (3)

where *W*_1_ and *W*_2_ are the dry weight of the membrane before and after alkali treatment; and *W*_3_ and *W*_4_ are the dry weight of the membrane before and after acid treatment, respectively.

#### 2.4.5. Eco-Efficiency

The eco-efficiency calculation is represented by conceptual Equation (4), which serves as a fundamental framework for quantifying environmental and economic performance. This equation encapsulates the essential components necessary for a comprehensive assessment of eco-efficiency, providing a robust analytical tool for researchers and practitioners in the field of sustainable development and environmental management.

(4) $$\mathrm{Eco}\text{-}\mathrm{efficiency}=\frac{\mathrm{value}\mathrm{of}\mathrm{the}\mathrm{product},\;\mathrm{process},\;\mathrm{or}\mathrm{service}\;(\mathrm{PPS})}{\mathrm{Environmental}\mathrm{impact}\mathrm{of}\mathrm{PPS}}$$

In this comprehensive study, the combination of sustainability and eco-efficiency prompted a change to the known eco-efficiency equation in (5). To appropriately depict the PPS, CO_2_/CH_4_ selectivity was used as a critical parameter. The fixed operating costs were thoroughly calculated, including those directly connected to membrane production. Concurrently, the operating cost included expenses for the gas mixture and energy usage within the separation unit. This method enables a full and accurate assessment of the system’s economic and environmental performance.

(5) $$\mathrm{Eco}\text{-}\mathrm{efficiency}=\frac{E_{d}}{E_{c}}$$

where *E_d_* is the CO_2_/CH_4_ selectivity, and *E_c_* is the fixed and operating costs.

### 2.5. CO_2_ Separation Using BC and CA Membranes

**Figure 2:**
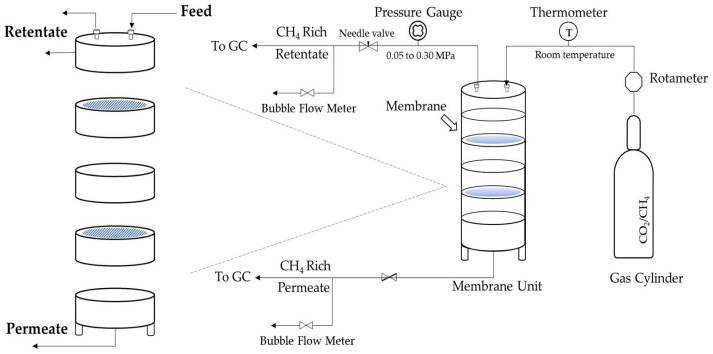
Membrane separation unit.

As seen in Figure 2, a membrane separation unit was developed to evaluate the BC and CA membranes’ capacity to separate CO_2_. This aluminum-constructed unit has five levels. The feed inlet and retentate outlet were both connected to one end of the apparatus. Between the feed and permeate chambers was a porous stainless-steel disc on which the membranes were placed. On the permeate chamber side, the permeate outlet was linked to the endplate. To prevent gas leaks, rubber O-rings were installed in each compartment. The flow rate of the gas mixture was controlled by a regulator located on top of the membrane separation unit. A rotameter was used to monitor and control the gas flow rate. The gas mixture of 40/60% CO_2_/CH_4_ was utilized to test the CO_2_/CH_4_ selectivity of the BC and CA membranes in the separation unit. The separation conditions were set following the suggestions from DE V.13: 30 °C temperature; and membrane thicknesses of 0.04 ± 0.005, 0.04 ± 0.005, and 0.05 ± 0.005 mm. Agilent gas chromatography technology (GC model 7890B) was used to examine the gas composition of the permeate and retentate streams.

The performance of CO_2_ separation from binary mixed gas was evaluated in terms of CH_4_ and CO_2_ component permeability and CO_2_/CH_4_ selectivity. The permeability of each component in a mixed gas system can be calculated with Equation (6) [15].

(6) $$P_{i}=\frac{Q*X_{perm,i}*l}{A(P_{feed}X_{feed,i\;-\;P_{perm}X_{perm,i}})}$$

where *P_i_* is the permeability of component *i*; *Q* is the feed flowrate; *X_perm,i_* is the mole fraction of component *i* in the permeate stream, *X_feed,i_* is the mole fraction of component *i* in the feed stream; *l* is the membrane thickness; and *A* is the membrane area.

The separation capability, quantified by the selectivity factor (*S_a/b_*), is expressed by Equation (7). This fundamental parameter plays a crucial role in chromatographic analysis and is essential for evaluating the efficiency of separation techniques. The selectivity factor, a dimensionless quantity, provides a quantitative measure of the relative retention of two analytes in a given chromatographic system.

(7) $$S_{a/b}=\frac{P_{a}}{P_{b}}$$

where *P_a_* is the permeability of component *a*, and *P_b_* is the permeability of component *b*.

## 3. Results

### 3.1. Characterization of BC and CA Membranes

Optimal cultivation conditions for BC and CA membrane production were identified using the conditions of pH 6, cultivation time of 10 days, and temperature of 30 °C. These parameters were maintained during BC cultivation from CW. Bio-CA membranes were created via acetylation of BC, with thicknesses of 0.03 ± 0.005, 0.04 ± 0.005, and 0.05 ± 0.005 mm. During the initial phase of cultivation, a thin, hydrated, gel-like BC membrane was observed to form. As cultivation progressed and the nutrient medium was progressively consumed, the membrane exhibited increased density and thickness. This observation provides strong evidence that CW serves as an efficacious carbon source for both BC and CA production. Furthermore, *A. Xylinum* demonstrated high efficiency in BC formation, effectively converting glucose derived from CW into biomass and BC. These findings underscore the potential of CW as a valuable substrate in microbial cellulose production processes. The visual characteristics of BC and CA membranes exhibit distinct differences. BC membranes present a brown coloration, while CA membranes display a predominantly white appearance. Furthermore, the tactile properties of these membranes differ significantly when they are dry. BC membranes possess a rubbery texture, in contrast to the crisp nature of CA membranes. These distinguishing features are important considerations in the selection and application of these materials for various industrial and research purposes.

#### 3.1.1. Morphological and Structural Analysis of BC and CA Membrane

**Figure 3:**
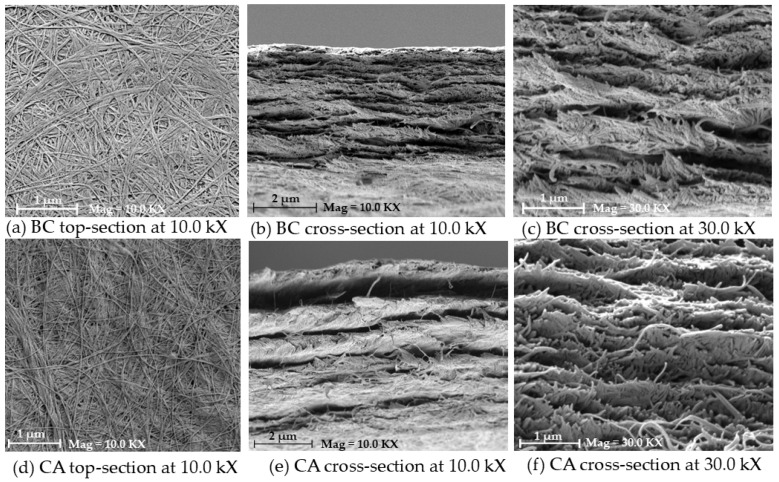
SEM results of the BC and CA membrane sample of (**a**,**d**) surface, (**b**,**e**) cross-section at 10 kx magnification, and (**c**,**f**) at 30 kx magnification.

The SEM results are displayed in Figure 3 for the structural and morphological characterization of membranes with different microstructures. Both BC and CA have an interwoven nanofiber network (Figure 3a,d), but BC’s fibrillar network is more uniform and denser than CA’s, which seems more erratic and loosely packed. The cross-sectional views of the images (Figure 3b,c,e,f) reveal that the CA membrane has a more porous and irregular lamellar structure, whilst the BC membrane has a layered and densely packed pattern with fewer pores. According to earlier research, the BC membrane’s tight and dense fibrillar structure suggests that it has the potential for greater filtration capabilities, high mechanical strength, and thermal stability [16]. Consistent with its uses in gas separation and dialysis membranes, the CA membrane’s more porous and loosely packed structure may lead to reduced mechanical strength but enhanced flexibility and permeability [17]. Because of their more compact design, BC membranes typically perform better in terms of durability and mechanical robustness when compared to other equivalent works, whereas CA membranes have advantages when higher permeability is required. Therefore, the unique application determines which of BC and CA is best: BC is appropriate for high-strength filtration systems, while CA is beneficial for separation.

#### 3.1.2. Chemical Analysis Structure by ATR-FTIR

**Figure 4:**
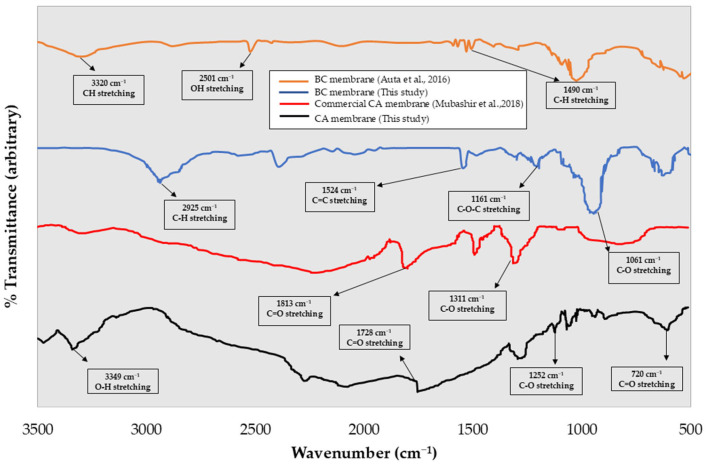
ATR-FTIR analysis of BC and CA membrane [20,21].

Figure 4 shows the ATR-FTIR analysis for both BC and CA membranes. Both bio-membranes have the common characteristics of the cellulose and cellulose acetate materials. The ATR-FTIR spectra showed a sharp peak at 2925 cm^−1^ corresponding to C-H stretching in the cellulose backbones. Other peaks at 1654 cm^−1^, 1314 cm^−1^, and 1161 cm^−1^, associated with C-O-C asymmetric stretching, were also observed [12,18]. In CA samples, a peak at 1210–1150 cm^−1^ notably appeared, related to C-C-O stretches in saturated esters of the cellulose acetate. These results are consistent with earlier studies on BC and commercial CA membranes [19].

#### 3.1.3. Tensile Strength of BC and CA Membrane

**Figure 5:**
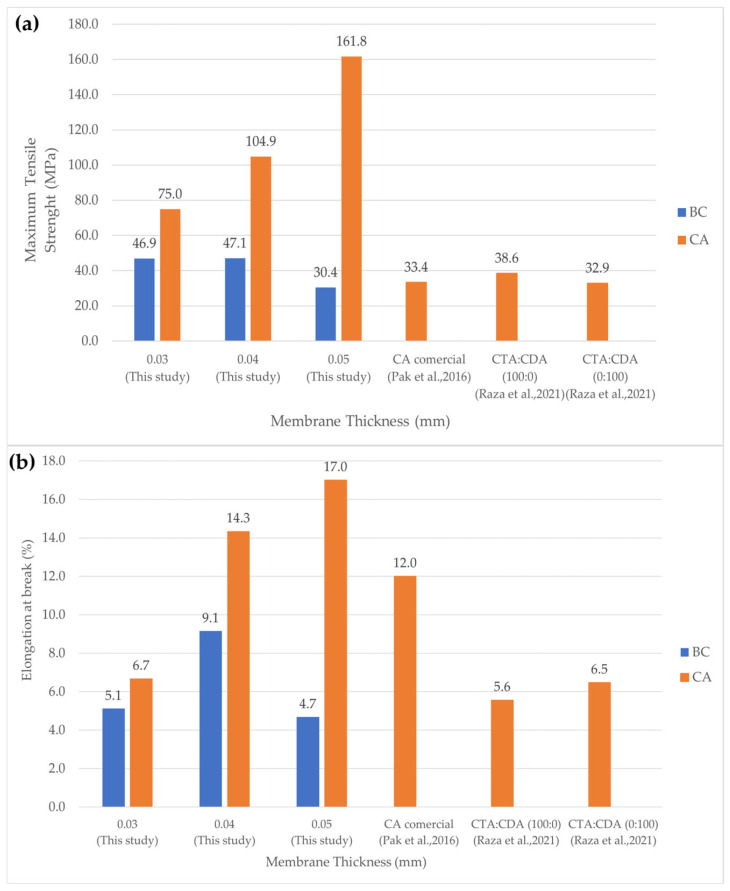
Tensile properties of BC and CA membranes: (**a**) tensile strength and (**b**) elongation at break [22,23].

Figure 5a presents the tensile properties of the sample membrane, with average tensile strengths ranging from 30.4 to 47.1 MPa and from 75.0 to 161.8 MPa for BC and CA membranes, respectively. Previous work [12] reported that the average tensile strength was in the range of 32.9–38.6 MPa. When tensile stress was applied to the membranes at room temperature, they exhibited semi-ductile properties. The samples had an average elongation at break of approximately 4.7–9.1% for the BC membrane and 6.7–17.0% for the CA membrane, as shown in Figure 5b. The CA-0.03 membrane (0.03 mm thick) demonstrated the highest tensile strength at around 161.8 MPa. Thicker membranes (0.04 and 0.05 mm) showed more pronounced fiber orientation under tensile stress, but the increased thickness likely caused slippage between fibrous layers under shear stress, leading to reduced strength. The membranes produced through the bioprocess in this study exhibited superior tensile strength compared to the unaltered membrane using the solvent-casting method for the CA solution. Notably, this enhanced structural integrity was achieved without the need for additional reinforcement, demonstrating the efficacy of the bioprocess approach in membrane synthesis. The CA membrane exhibits superior tensile strength in comparison to the BC membrane, attributable to the acetylation process involving the addition of acetyl groups to the membrane structure. This modification enhances the interaction between the polymer and plasticizers, resulting in improved mechanical properties. Research has demonstrated that acetylated citrates display enhanced plasticizing efficiency, leading to increased tensile strength and elongation at break relative to their non-acetylated counterparts [19]. The CA membrane synthesized in this study demonstrates higher tensile strength compared to the commercial CA membrane reported by [22] and is higher than that of CTA/CA (100:0) and CTA/CA (0:100) reported by [23]. Moreover, cross-linked CA membranes have been shown to possess tensile strength four times that of standard CA membranes, highlighting the potential for enhanced mechanical properties through chemical modifications [24]. The incorporation of lignin into BC and CA significantly enhances the tensile strength of cellulose-based composites. Lignin acts as a filler and functional agent, improving the mechanical properties of BC by 142% in strength and 63% in toughness when optimally impregnated [25].

#### 3.1.4. Membrane Composition Analysis

**Figure 6:**
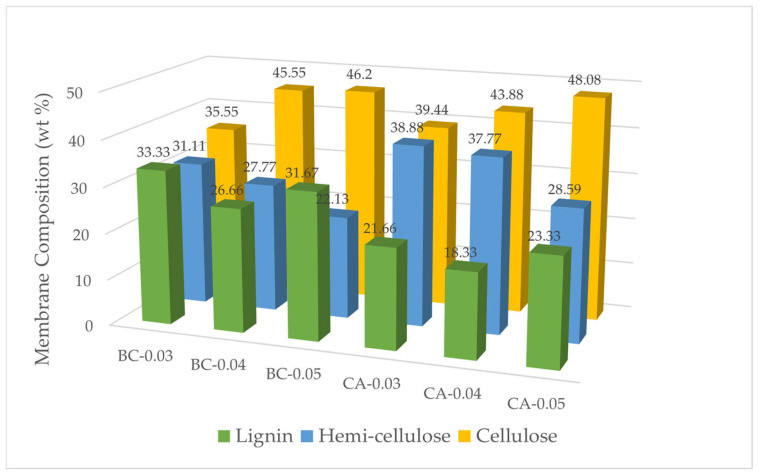
BC and CA membrane composition.

Figure 6 provides a comprehensive summary of the compositional variations in BC and CA membranes at thicknesses of 0.03, 0.04, and 0.05 mm, with a focus on the proportions of cellulose, hemicellulose, and lignin. In the BC membranes, the cellulose content increases as the thickness increases, from 35.55% at 0.03 mm to 46.20% at 0.05 mm, indicating a denser cellulose network in thicker membranes. The CA membranes show more variability, with a cellulose content reaching 48.08% at 0.05 mm, a marked increase compared to thinner samples. Interestingly, the hemicellulose content in BC decreases with increasing thickness, from 31.11% to 22.13%, while lignin levels also show variability. In comparison, CA membranes exhibit a high hemicellulose content in thinner samples, peaking at 38.88% at 0.03 mm, and decreasing with increasing thickness. Lignin content in both BC and CA remains relatively consistent but shows a slight decline in thicker membranes. When compared with previous studies, the findings demonstrate a typical range for cellulose content in BC membranes, where similar research by [26] reported cellulose content in BC membranes to be within the range of 40–50% depending on growth conditions and thickness, aligning well with the data presented. However, the hemicellulose and lignin contents observed here are higher than those reported in [27], where BC membranes typically showed hemicellulose content below 25%, likely due to differences in production methods. For CA membranes, the compositional variability is significant compared to previous research. It has been reported that CA membranes have cellulose content as high as 55% [28], especially in membranes used for high-performance applications, a value slightly higher than what was observed in this study’s thickest CA sample (48.08%). The high hemicellulose content in the thinner CA samples (47.77%) matches well with the authors of [29], as they also observed that thinner CA membranes tend to retain more hemicellulose due to less processing required during preparation. However, the lignin content is higher than typically reported in both CA and BC membranes, in comparison with [30], which found lignin content in CA membranes to be less than 25%, suggesting that the materials used in this study might contain different precursor materials or be subjected to fewer purification steps. Overall, the results suggest that thicker BC and CA membranes exhibit higher cellulose content, aligning with their potential for more durable applications, while thinner membranes with higher hemicellulose content might be better suited for applications requiring flexibility and permeability. The variability in composition compared to other works also highlights the influence of processing techniques and materials used on membrane composition.

### 3.2. CO_2_ Separation Performance of the BC and CA Membrane from CO_2_/CH_4_ Mixed Gas

The CO_2_/CH_4_ separation performance of both BC and CA membranes was determined by measuring the CO_2_/CH_4_ selectivity on the permeate side. The compositions of CH_4_ and CO_2_ of the permeate and retentate were determined from gas chromatography results. The permeability and selectivity were then calculated from the composition of the gas components. Table 1 presents a summary of the gas permeation results.

**Figure 7:**
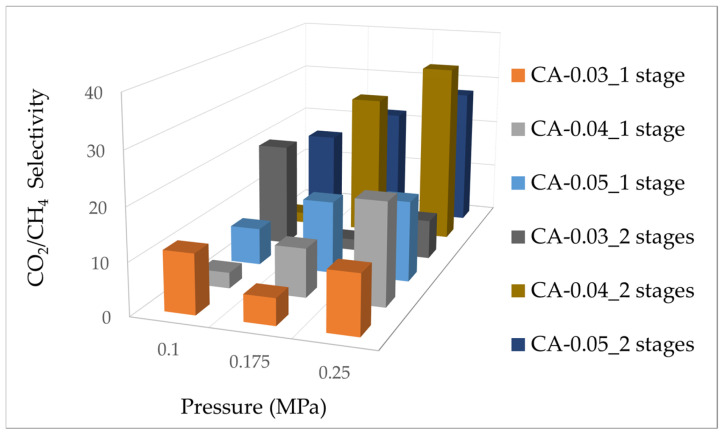
CO_2_/CH_4_ selectivity vs. pressure (MPa) of CA membrane.

The effect of feed pressure on CO_2_/CH_4_ selectivity using CA membrane separation was explored in this study, highlighting the role of the number of stages in the separation unit. Figure 7 shows the CO_2_/CH_4_ selectivity values of the CA membranes of 0.03, 0.04, and 0.05 mm in thickness under various operating feed pressures (0.1, 0.175, and 0.25 MPa) in the membrane separation unit (single or double stage configuration). From the graphs, we can see that the separation performance improved, as the CO_2_ selectivity was likely to increase with the increasing pressure. CO_2_ has more solubility inside the CA matrix than CH_4_ when both components diffuse across the membrane [10]. Furthermore, higher selectivity values were significantly achieved in the double-stage separation, particularly at high pressure (for example, 0.175 and 0.25 MPa), likely due to increased membrane surface area. A more pronounced improvement in selectivity was observed at a higher feed pressure, especially at 0.25 MPa, compared to its single tray configuration. In general, thicker membranes were likely to have better tolerance against high pressure since they have superior mechanical strengths, resulting in relatively stable selectivity across the tested pressure range. For CA membranes, the CO_2_/CH_4_ selectivity increased with reduced membrane thickness, as thinner membranes minimized resistance to CO_2_ transport, allowing for enhanced permeability and improved separation performance. This trend was observed consistently across both single-stage and double-stage configurations [31,32].

**Figure 8:**
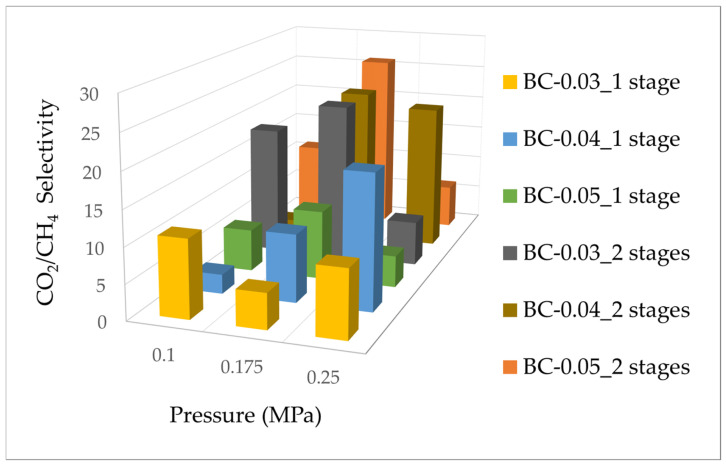
CO_2_/CH_4_ selectivity vs. pressure (MPa) of BC membrane.

Figure 8 shows the selectivity response of BC membranes under varying pressures, tested in both single- or double-stage configurations for membrane samples with different thicknesses: BC-0.03 (0.03 mm), BC-0.04 (0.04 mm), and BC-0.05 (0.05 mm). Selectivity is plotted as a function of applied pressure, ranging from 0.1 to 0.25 MPa. In the single tray setup, BC-0.03 shows a significant increase in selectivity as pressure increases to 0.175 MPa, followed by a sharp decline at 0.25 MPa. BC-0.04 initially demonstrates a similar trend, with selectivity peaking at 0.175 MPa before decreasing at higher pressures. The experimental results indicate distinct pressure-dependent behaviors among the BC membrane variants. BC-0.05 exhibits a more gradual increase in selectivity at lower pressures and maintains relatively consistent performance across the higher-pressure range. In contrast, BC-0.03 and BC-0.04 demonstrate optimal selectivity at moderate pressures. These observations suggest that each BC membrane responds uniquely to pressure variations. In the double-stage configuration, BC-0.03 demonstrates higher initial selectivity at 0.1 MPa, reaching a pronounced peak at 0.175 MPa, followed by a decline at 0.25 MPa. This pattern indicates optimal performance at mid-range pressures. BC-0.04 exhibits a steady increase in selectivity up to 0.175 MPa, maintaining a consistent level at 0.25 MPa. BC-0.05, however, displays a modest increase in selectivity as pressure rises, with less fluctuation between pressure points compared to the other samples. It is noteworthy that despite these variations in performance among the BC membrane variants, the CO_2_ separation capability of BC was generally inferior to that of CA. These findings provide valuable insights into the pressure-dependent behavior of BC membranes and their relative performance compared to CA in CO_2_ separation applications. Thinner membranes demonstrated higher selectivity due to reduced mass transfer resistance, which facilitated greater CO_2_ permeability, and also maintained superior selectivity compared to thicker ones, as they were better able to exploit the increased CO_2_ solubility without compromising the separation efficiency due to CH_4_ transport [31].

**Figure 9:**
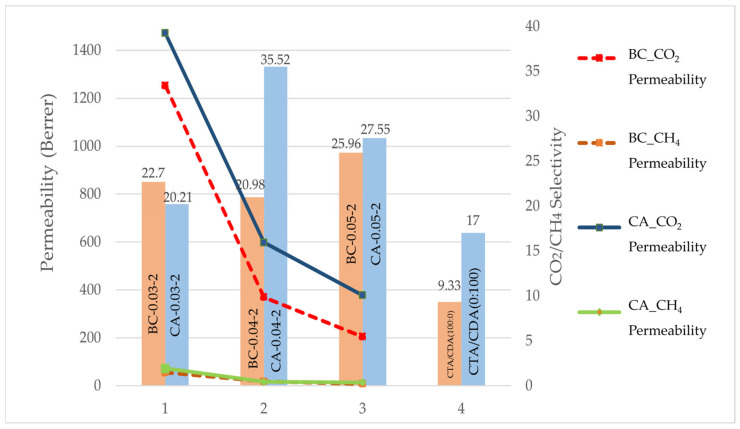
Separation performance of BC and CA membranes.

Figure 9 illustrates the relationship between CO_2_/CH_4_ selectivity and membrane thickness for CA and BC membranes at the double stage. At a thickness of 0.03 mm, the BC membrane demonstrates slightly higher CO_2_/CH_4_ selectivity, with a value of 22.7 compared to 20.21 for the CA membrane. This suggests that with thinner membranes, the BC performs slightly better in terms of CO_2_/CH_4_ selectivity. However, at a thickness of 0.04 mm, a significant change is observed. The CA membrane exhibits a much higher CO_2_/CH_4_ selectivity, with a value of 35.52, far surpassing BC’s selectivity of 20.98 at the same thickness. This indicates that CA membranes achieve their highest CO_2_/CH_4_ selectivity at this particular thickness, significantly outperforming BC. As the thickness increases to 0.05 mm, both membranes show similar performance. The CO_2_/CH_4_ selectivity of the BC membrane is 25.96, while that if the CA membrane is slightly higher, at 27.55. This suggests that for thicker membranes, the selectivity difference between the two materials becomes lower, with CA having slightly more than BC. Overall, the data suggest that while BC performs better at the thinnest membrane (0.03 mm), CA shows superior CO_2_/CH_4_ selectivity at 0.04 mm and continues to perform slightly better at 0.05 mm. The optimal performance for CA occurs at a membrane thickness of 0.04 mm, where it shows a significant increase in selectivity compared to BC. Ref. [23] reported the selectivity of CTA/CDA (100:0) and CTA/CDA (0:100) at 9.33 and 17, respectively. The observed reduction in performance can be attributed to the superior CO_2_/CH_4_ selectivity exhibited by bio-CA membranes in comparison to their blended counterparts. This enhanced selectivity is primarily a result of the bio-based structure’s capacity to incorporate a higher density of functional sites, which preferentially facilitate CO_2_ transport over CH_4_. Consequently, in applications where selectivity takes precedence over permeability, bio-CA membranes emerge as the optimal choice. The CO_2_ removal mechanisms in BC and CA membranes differ significantly due to their structural and chemical properties. BC membranes, when modified, exhibit high hydroxide-ion conductivity and Faradaic efficiency for CO_2_ electrochemical reduction, indicating a strong interaction with polar species like hydroxide ions, facilitating CO_2_ capture and conversion [33,34]. In contrast, CA membranes, particularly those blended with ionic liquids, demonstrate enhanced CO_2_ solubility due to the polar interactions between the ionic liquid and the carbonyl groups of CA, improving CO_2_ affinity [35]. Additionally, CA membranes show significant selectivity for CO_2_ over methane, attributed to their solubility selectivity, while BC membranes focus on ionic transport and electrochemical processes [36]. Thus, the polar nature of BC enhances its electrochemical performance, while CA’s polar interactions improve gas solubility, leading to effective CO_2_ removal in different mechanisms [35,37].

**Figure 10:**
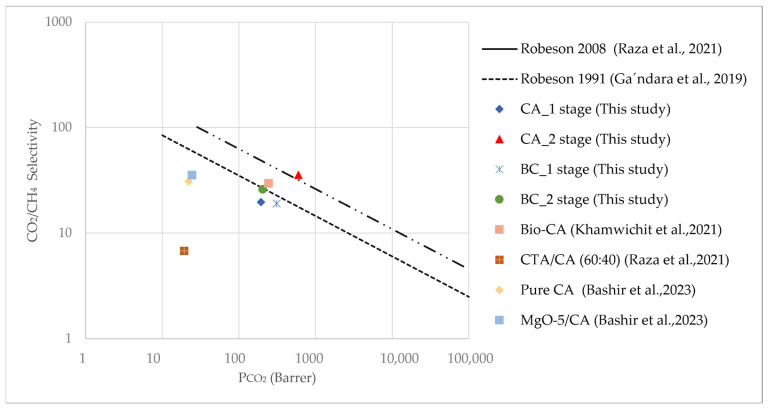
CO_2_/CH_4_ gas separation performance of various BC and CA membranes in comparison to the Robeson upper bound [12,23,38,39].

The membrane performance results of this study show that both CA and BC membranes exhibit competitive CO_2_/CH_4_ separation efficiency, with performance lying between Robeson’s 1991 and 2008 upper bounds, as seen in Figure 10. Notably, the CA membranes, particularly in the double-stage configuration, offer a promising balance of permeability and selectivity, outperforming recent alternatives such as Bio-CA membranes [12] and CTA/CDA blends [23]. The two concurrent stage setups provide increased selectivity at the expense of permeability, as expected for the procedures. While these membranes do not yet exceed the Robeson (2008) upper bound, their proximity to it suggests that further optimization in material properties and design could improve both selectivity and permeability, potentially leading to breakthroughs in membrane technology for gas separations.

### 3.3. Optimization of CO_2_ Removal Using Membrane Separation Units

Table 1 lists both BC and CA membranes for 26 experiments designed by DE V.13. Statistical optimization through experimental design is a methodology that enables the identification of optimal conditions in diverse scenarios. This approach systematically establishes the intricate relationships between various factors. By leveraging this advanced technique, the most favorable operating conditions, thereby maximizing process efficiency and environmental sustainability, can be determined. As seen in the table, the highest CO_2_/CH_4_ selectivity and eco-efficiency values of 35.52 and 2.32 CO_2_/CH_4_ selectivity/THB were obtained at the gas separation conditions of pressure at 0.281 MPa and CA thickness of 0.04 mm in two-stage separation units.

#### 3.3.1. Statistical Analysis for CO_2_/CH_4_ Selectivity

The model summary statistics for CO_2_/CH_4_ selectivity, as shown in Table 2, indicate that the coefficient of determination (R^2^) of the proposed model is better than 0.9, indicating a strong fit. By maximizing the adjusted R^2^, the suggested Quartic model is identified as the best fit for predicting CO_2_/CH_4_ selectivity. The ANOVA results, presented in Table 2, confirm the model’s significance, with an F-value of 525.77, indicating a highly significant model. A *p*-value less than 0.0500 indicates that a model term is significant. In this case, the terms A (gas pressure), B (membrane thickness), C (number of stages), AB, AC, BC, A^2^, B^2^, ABC, A^2^B, A^2^C, AB^2^, B^2^C, A^2^B^2^, and AB^2^C are all significant. The intercept values (18.74 for selectivity and 2.21 for eco-efficiency) represent the baseline responses when all variables are at their center points. The values for A (8.86005, 0.542056), B (5.26796, 0.638295), and C (9.345, 0.876135) show their individual effects. Positive coefficients indicate a direct relationship, where increasing these factors improves the responses. The interaction coefficients, such as AB (3.88375, 0.312043), AC (2.96278, 0.174402), and BC (1.72534, 0.148463), represent how pairs of variables interact. For example, a positive AB coefficient means that the combined increase in A and B enhances the response. Conversely, negative values of quadratic terms (A^2^ and B^2^) denote diminishing returns to the responses as these factors reach higher levels. Such as the coefficients for CO_2_/CH_4_ selectivity (−1.8275 and −4.08502) and eco-efficiency (−0.4971 and −0.4671) indicate complex relationships between variables. Higher-order and mixed interaction coefficients, such as ABC and A^2^B, account for complex interactions among three variables or non-linear contributions. For instance, the coefficient for ABC (2.17625, 0.138263) indicates a significant three-factor interaction, while terms like A^2^B (1.57127, 0.114782) highlight the combined effect of non-linear A with linear B, as reported in Equations (8) and (9).

CO_2_/CH_4_ selectivity = 18.75 + 8.86A + 5.27B + 9.34C + 3.88AB + 2.96AC + 1.73BC − 1.83A^2^ − 4.09B^2^ + 2.18ABC − 3.53A^2^B − 2.62A^2^C − 8.12AB^2^ − 3.71B^2^C + 1.57A^2^B^2^ + 0.4134A^2^BC − 3.90AB^2^C (8)

Eco-efficiency = 2.21 + 0.5421A + 0.6383B + 0.8761C + 0.3120AB + 0.1744AC + 0.1485BC − 0.4971A^2^ − 0.4671B^2^ + 0.1383ABC − 0.5262A^2^B − 0.3591A^2^C − 0.6013AB^2^ − 0.3942B^2^C + 0.1148A^2^B^2^ + 0.0464A^2^BC − 0.2804AB^2^C (9)

where A is pressure (MPa), B is thickness (mm), and C = number of separation stage.

**Figure 11:**
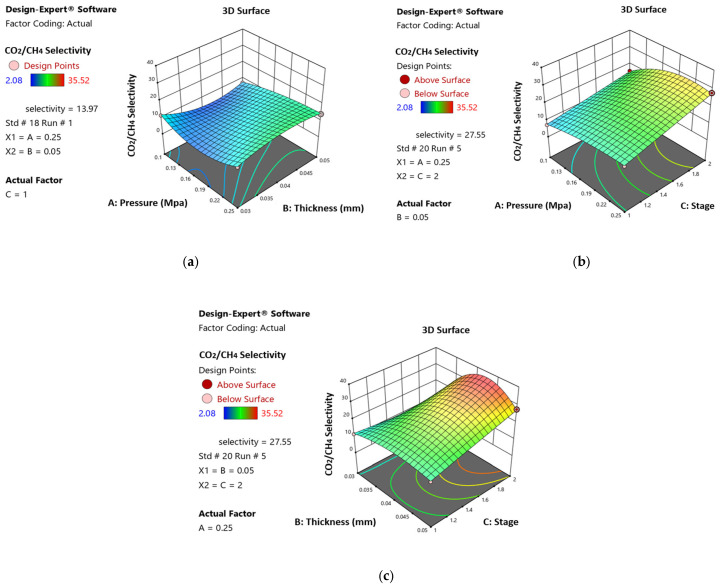
Response surface plot demonstrates the interaction effect of (**a**) pressure and thickness, (**b**) pressure and stage, and (**c**) thickness and stage for CO_2_/CH_4_ selectivity.

These diagrams can be used to identify the best parameter combinations and clarify complex relationships that might not be immediately clear using more conventional analytical techniques. The results of the interaction effects between the three independent parameters, which were pressure, thickness, and stage, on the dependent parameter, CO_2_/CH_4_ selectivity, are depicted in Figure 11. Figure 11a demonstrates the interaction between pressure and thickness, highlighting their significant combined effect on CO_2_/CH_4_ selectivity within the examined range. Similarly, Figure 11b illustrates the interaction between pressure and stage, where the relationship between these variables plays a crucial role in influencing CO_2_/CH_4_ selectivity. Lastly, Figure 11c shows the interaction between thickness and stage, revealing how variations in these parameters jointly affect CO_2_/CH_4_ selectivity. These surface diagrams provide valuable insights into the interplay of independent variables, allowing for a more precise adjustment of experimental conditions to achieve optimal CO_2_/CH_4_ selectivity.

The optimization of CO_2_/CH_4_ selectivity was achieved through the application of DE V.13. A best-fitted model, derived from experimental data, was in the form of a quartic equation. The resultant equation, presented as Equation (8), provides a robust framework for predicting optimal conditions. This methodological approach ensures a high degree of accuracy and reliability in determining the most favorable parameters for maximizing selectivity in the CO_2_/CH_4_ system.

#### 3.3.2. Statistical Analysis of Eco-Efficiency

In this study, the overall cost of producing BC and CA membranes from CW was estimated as follows: The overall production cost of BA and CA membranes is 0.42 and 1.86 THB, respectively. The operational cost of membrane separation testing in this study, determined based on the gas mixture used in each experiment, was 5.10 THB/Litter. Table 1 shows the eco-efficiencies of 26 experiments. The results show that the eco-efficiency was influenced by the number of stages, which is the most important variable influencing the CO_2_/CH_4_ selectivity and total cost. Furthermore, membrane thickness did not drastically affect the cost of preparing CA membranes. While thicker membranes may provide better CO_2_ separation performance than thinner membranes, thicker membranes are more cost-effective for prospective CO_2_-removal applications. The results show that the BC membrane with a thickness of 0.03 mm at an air inlet pressure of 0.1 MPa and the CA membrane with a thickness of 0.04 mm and a pressure of 0.175 MPa with a double stage of tray provided the highest eco-efficiency at 5.04 and 3.08 CO_2_/CH_4_ selectivity/THB.

The model summary statistics for eco-efficiency, as presented in Table 2, demonstrate a robust coefficient of determination (R^2^) exceeding 0.9 for the proposed model. This high R^2^ value indicates a strong explanatory power and fitting, lending substantial reliability to the model’s predictive capabilities in the context of eco-efficiency analysis. The model’s significance was assessed using the ANOVA, and the findings are shown in Table 2. The Model F-value of 187.71 demonstrates the model’s significance in terms of eco-efficiency, and *p*-values of less than 0.0500 suggest that model terms are significant. Significant model terms include A, B, C, AB, AC, BC, A^2^, B^2^, and ABC. Values above 0.1000 imply that the model terms are not significant.

**Figure 12:**
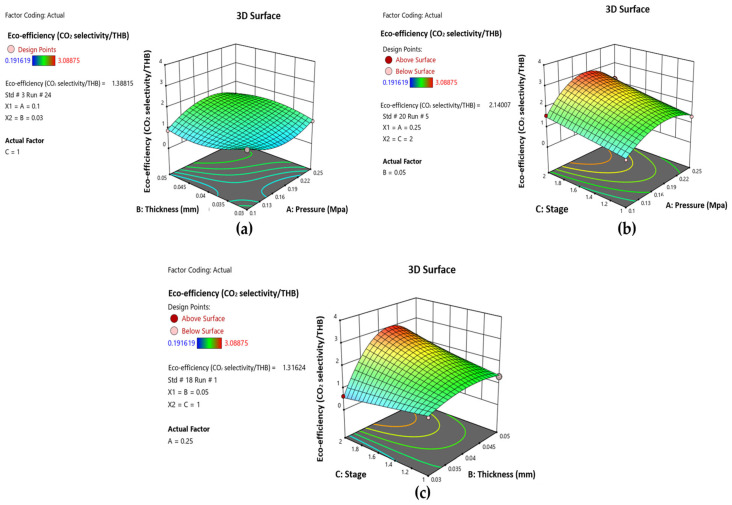
Respond surface plot demonstrates the interaction effect of (**a**) pressure and thickness (**b**) pressure and stage and (**c**) thickness and stage for eco-efficiency.

The regression equation can be seen visually in a three-dimensional surface diagram, allowing for comprehensive analysis and interpretation of complex statistical relationships. Figure 12 depicts the interaction effects of the three independent parameters on the dependent parameter, eco-efficiency. Figure 12a depicts the interplay of pressure and thickness, emphasizing their large combined effect on eco-efficiency within the studied range. Similarly, Figure 12b displays the interaction between pressure and stage, demonstrating how the link between these variables influences eco-efficiency. Finally, Figure 12c illustrates the correlation between thickness and stage, demonstrating how changes in these factors affect eco-efficiency. These surface diagrams provide useful insights into the interaction of independent factors, allowing for more accurate adjustment of experimental conditions to attain maximum eco-efficiency. Model optimization from DE V.13 was conducted to establish the best settings for optimizing eco-efficiency. The best-fitted model was obtained by deriving a quartic equation from the experimental data. The equation is shown in Equation (9).

### 3.4. Validation of CO_2_/CH_4_ Selectivity and Eco-Efficiency

To verify the fitting model developed from DE V.13 optimization to predict CO_2_/CH_4_ selectivity and eco-efficiency, a series of experiments were carried out. As seen from Table 3, the values of CO_2_/CH_4_ selectivity and eco-efficiency obtained from the experiment are in good agreement with those from the model. The outcomes validate the model’s outstanding prediction for CO_2_/CH_4_ selectivity and eco-efficiency. The predicted mean closely aligns with the actual values, indicating the reliability of the model. Standard deviations and standard errors of prediction are minimal, reflecting consistent accuracy across predictions. The 95% prediction intervals (PI) provide a range within which future observations are expected to fall, showcasing the robustness of the model under varying conditions. Overall, the close agreement between actual and predicted data underscores the model’s validity for optimizing performance.

### 3.5. Optimization Conditions of CO_2_/CH_4_ Selectivity and Eco-Efficiency

**Figure 13:**
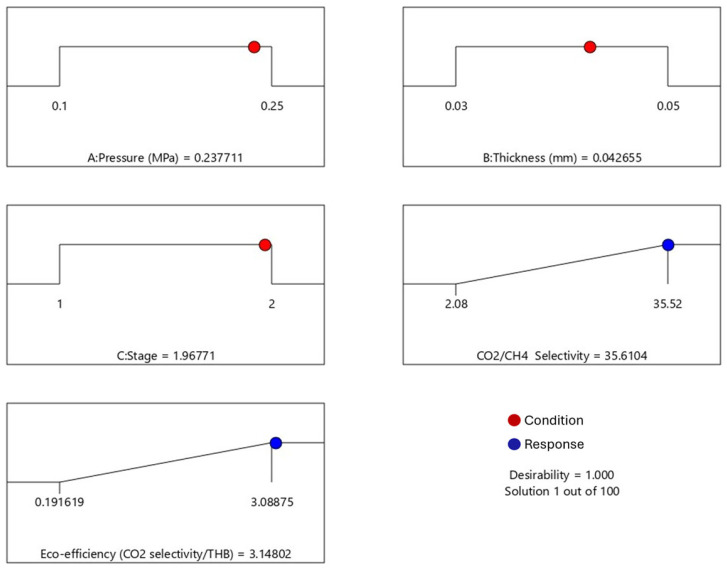
Desirability ramps of CO_2_/CH_4_ selectivity and eco-efficiency.

The response optimizer function in the DE was utilized to optimize CO_2_/CH_4_ selectivity and eco-efficiency parameters. The optimal conditions were determined based on the highest value of the composite desirability (D) function, which ranges from 0 to 1. A D value of 1 indicates complete satisfaction with the criteria [40]. From Figure 13, we can see that the optimal conditions yielding the maximum CO_2_/CH_4_ selectivity and eco-efficiency of 35.61 and 3.148, respectively, were at a gas pressure of 0.23 MPa, a membrane thickness of 0.04 mm, and two stages of gas separation units.

## 4. Conclusions

This study successfully demonstrates the potential of BC and CA membranes synthesized from CW for effective CO_2_/CH_4_ gas separation. This research highlights that membrane thickness, pressure, and the number of separation stages are critical factors influencing both CO_2_ selectivity and eco-efficiency. The CA membranes, particularly at 0.04 mm thickness and in a double-stage configuration, exhibited the highest CO_2_/CH_4_ selectivity of 35.52, surpassing BC membranes in performance. Additionally, the findings emphasize that CA membranes are more suitable for industrial applications requiring higher mechanical strength and gas selectivity, while BC membranes offer flexibility in thinner formats. The eco-efficiency analysis further indicates that thicker membranes, though more cost-effective for CO_2_ separation, still require optimization for operational cost reduction. The maximum eco-efficiency of 3.08 CO_2_/CH_4_ selectivity/THB was achieved. Overall, the study confirms that bio-based membranes, especially CA, represent a promising, sustainable alternative for industrial gas separation processes, contributing to the development of more environmentally friendly solutions for biogas upgrading and natural gas purification. This study introduces the innovative application of CA membranes with optimized thickness and multi-stage configurations, achieving unprecedented CO_2_/CH_4_ selectivity while maintaining eco-efficiency.

## Figures and Tables

**Table 1:** CO_2_/CH_4_ selectivity and eco-efficiency of CA membranes.

	Factor 1	Factor 2	Factor 3	Response 1	Response 2
Run	A: Pressure (MPa)	B: Thickness (mm)	C: Stage	CO_2_/CH_4_ Selectivity	Eco-Efficiency CO_2_/CH_4_ Selectivity/THB
1	0.250	0.050	1	15.62	1.47
2	0.100	0.030	2	20.21	1.85
3	0.175	0.040	2	28.09	3.08
4	0.175	0.025	1	4.05	0.55
5	0.25	0.050	2	27.55	2.14
6	0.175	0.040	1	9.40	1.33
7	0.175	0.040	2	28.09	3.08
8	0.175	0.040	1	9.40	1.33
9	0.175	0.054	1	14.07	1.94
10	0.175	0.040	2	28.09	3.08
11	0.175	0.040	1	9.40	1.33
12	0.175	0.040	2	28.09	3.08
13	0.281	0.040	2	35.52	2.32
14	0.175	0.054	2	21.98	2.41
15	0.060	0.040	1	3.04	0.60
16	0.175	0.040	2	28.09	3.08
17	0.281	0.040	1	19.72	1.64
18	0.100	0.050	1	7.20	0.87
19	0.175	0.025	2	2.20	0.19
20	0.068	0.040	2	2.08	0.30
21	0.250	0.030	1	11.36	1.13
22	0.250	0.030	2	7.68	0.62
23	0.175	0.040	1	9.40	1.33
24	0.100	0.030	1	11.42	1.38
25	0.175	0.040	1	9.40	1.33
26	0.100	0.050	2	15.84	1.57

**Table 2:** Model summary for CO_2_/CH_4_ selectivity and eco-efficiency of CA membranes.

Source	CO_2_/CH_4_ Selectivity		Eco-Efficiency	
	Sum of Squares	*p*-Values	Sum of Squares	*p*-Values
R^2^	0.99		0.99	
Adjusted R^2^	0.99		0.99	
Intercept	18.74		2.21	
Model	2415.45	<0.0001	20.53	<0.0001
A—pressure (MPa)	628.00	<0.0001	2.35	<0.0001
B—thickness (mm)	222.01	<0.0001	3.26	<0.0001
C—layer	873.29	<0.0001	7.68	<0.0001
AB	120.67	<0.0001	0.7790	<0.0001
AC	70.22	<0.0001	0.2433	0.0002
BC	23.81	<0.0001	0.1763	0.0007
A^2^	38.17	<0.0001	2.82	<0.0001
B^2^	190.71	<0.0001	2.49	<0.0001
ABC	37.89	<0.0001	0.1529	0.0011
A^2^B	49.89	<0.0001	1.11	<0.0001
A^2^C	95.25	<0.0001	1.79	<0.0001
AB^2^	263.98	<0.0001	1.45	<0.0001
B^2^C	191.92	<0.0001	2.16	<0.0001
A^2^B^2^	9.88	0.0002	0.0527	0.0215
A^2^BC	0.6836	0.1572	0.0086	0.2911
AB^2^C	60.97	<0.0001	0.3146	<0.0001

**Table 3:** Confirmation of condition optimization.

Analysis	Conditions			Actual	Predicted Mean	Std Dev	95% PI Low	95% PI High
	Pressure (MPa)	Thickness (mm)	Stages					
CO_2_ selectivity	0.1	0.03	2	21.15	19.808	0.536	18.121	21.495
Eco-efficiency	0.1	0.03	2	1.76	1.795	0.083	1.534	2.055
CO_2_ selectivity	0.175	0.04	2	28.50	28.090	0.536	26.762	29.418
Eco-efficiency	0.175	0.04	2	3.08	3.088	0.083	2.884	3.294
CO_2_ selectivity	0.25	0.04	2	34.98	35.468	0.536	34.030	36.908
Eco-efficiency	0.25	0.04	2	3.02	2.949	0.083	2.727	3.171
CO_2_ selectivity	0.175	0.05	2	26.63	27.284	0.536	25.845	28.723
Eco-efficiency	0.175	0.05	2	3.10	3.014	0.083	2.792	3.236
CO_2_ selectivity	0.25	0.03	2	8.91	7.278	0.536	5.591	8.965
Eco-efficiency	0.25	0.03	2	0.68	0.564	0.083	0.303	0.824

## Data Availability

The data and code that support the conclusions described in this research, as well as further discoveries from this investigation, are accessible from the corresponding author upon reasonable request.
